# The Role of IL-17A in Mediating Inflammatory Responses and Progression of Neurodegenerative Diseases

**DOI:** 10.3390/ijms26062505

**Published:** 2025-03-11

**Authors:** Miao-Yan Zheng, Lian-Zhong Luo

**Affiliations:** 1 School of Pharmacy, Fujian Medical University, Xuefu North Road 1, University Town, Fuzhou 350122, China; zmy@fjmu.edu.cn; 2 Fujian Universities and Colleges Engineering Research Center of Marine Biopharmaceutical Resources, Xiamen Medical College, 1999 Guankouzhong Road, Xiamen 361023, China

**Keywords:** IL-17A, Th17, neurodegenerative diseases, neuro-inflammation, multiple sclerosis, Alzheimer’s disease, Parkinson’s disease

## Abstract

IL-17A has been implicated as a critical pro-inflammatory cytokine in the pathogenesis of autoimmune and neurodegenerative disorders. Emerging evidence indicates its capacity to activate microglial cells and astrocytes, subsequently inducing the production of inflammatory mediators that exacerbate neuronal injury and functional impairment. Clinical observations have revealed a demonstrated association between IL-17A concentrations and blood–brain barrier (BBB) dysfunction, creating a pathological feedback loop that amplifies neuro-inflammatory responses. Recent advances highlight the cytokine’s critical involvement in neurodegenerative disorders through multiple molecular pathways. Therapeutic interventions utilizing monoclonal antibodies (mAbs) against IL-17A or its cognate receptor (IL-17R) have shown promising clinical potential. This review systematically examines the IL-17A-mediated neuro-inflammatory cascades; the mechanistic contributions to neurodegenerative pathology in the established disease models including multiple sclerosis, Alzheimer’s disease, Parkinson’s disease, and amyotrophic lateral sclerosis; and current therapeutic strategies targeting the IL-17A signaling pathways. The analysis provides novel perspectives on optimizing cytokine-directed therapies while identifying the key challenges and research priorities for translational applications in neurodegeneration.

## 1. Research Progress on Inflammation-Induced Neurodegenerative Diseases

### 1.1. Types of Neurodegenerative Diseases

Neurodegenerative disorders constitute a heterogeneous class of neurological conditions characterized by the progressive degeneration of selective neuronal populations, culminating in irreversible functional decline. These clinically heterogeneous entities manifest distinct neurological and symptomatologic profiles, encompassing prototypical conditions including Alzheimer’s disease (AD), Parkinson’s disease (PD), multiple sclerosis (MS), and amyotrophic lateral sclerosis (ALS) [1]. While demonstrating distinct etiological mechanisms, these disorders exhibit a shared pathophysiological feature of sustained neuro-inflammatory activation [2].

### 1.2. Impact of Inflammation on the Neural Microenvironment

Inflammation is a critical component of the central nervous system (CNS) microenvironment, playing a dual role in both protection and pathology [3]. Under normal physiological conditions, acute neuro-inflammation serves as a defense mechanism, facilitating the removal of pathogens, cellular debris, and damaged tissues [4,5]. However, when inflammation becomes chronic or dysregulated, it disrupts neural homeostasis, leading to neuronal dysfunction and contributing to the progression of neurodegenerative diseases [6,7]. Neuro-inflammation is critically influenced by glial cell activation, a cellular process vital for CNS homeostasis maintenance.

Microglia, the resident immune cells of the brain, are the primary mediators of neuro-inflammatory responses, rapidly responding to injury or infection by releasing cytokines and chemokines. Astrocytes, traditionally considered structural support cells, also actively participate in inflammation by modulating immune signaling, regulating synaptic activity, and maintaining the blood–brain barrier (BBB) [8]. In a healthy CNS, microglia and astrocytes collaborate to resolve inflammation and restore homeostasis. However, under chronic inflammatory conditions, these cells enter a state of sustained activation. This persistent activation amplifies neuro-inflammation and worsens neuronal damage [9,10,11].

Interleukin-17A (IL-17A), a key pro-inflammatory cytokine, has been increasingly recognized as a critical driver of glial cell activation in neurodegenerative diseases [12,13,14,15]. Studies show that IL-17A stimulates microglia and astrocytes to release neurotoxic mediators such as tumor necrosis factor-alpha (TNF-α), interleukin-1 beta (IL-1β), and interleukin-6 (IL-6), which further propagate inflammation [16]. Additionally, IL-17A compromises the integrity of the BBB by increasing endothelial permeability, allowing peripheral immune cells and inflammatory molecules to infiltrate the CNS. This leads to metabolic and structural alterations in the neural microenvironment, contributing to oxidative stress, synaptic dysfunction, and neuronal degeneration [17,18,19]. Therefore, understanding the role of IL-17A in regulating glial cell activity and its impact on the neural microenvironment is crucial for developing targeted therapies that aim to reduce neuro-inflammation and maintain neuronal function.

### 1.3. Studies on the Correlation Between Inflammation and Neurodegenerative Diseases

Neuro-inflammation plays a critical role in the onset and progression of various neurodegenerative diseases [2]. Among the key inflammatory mediators, IL-17A has gained increasing attention for its involvement in CNS pathology [20]. Studies have demonstrated that IL-17A can disrupt the BBB, activate glial cells, and promote neuronal damage, thereby accelerating neurodegenerative processes [21,22,23,24]. Elevated levels of IL-17A and its associated signaling pathways have been observed in patients with neurodegenerative diseases, suggesting a strong link between IL-17A-driven inflammation and disease progression. In MS, for example, IL-17A has been implicated in autoimmune-mediated demyelination, contributing to neuronal injury and functional impairment [25]. Similarly, in AD and PD, IL-17A has been associated with increased neuro-inflammatory responses, exacerbating neurodegeneration [26]. However, the precise mechanisms through which IL-17A contributes to these conditions remain an area of active research.

This review examines the relationship between neuro-inflammation and CNS neurodegenerative diseases, with a particular emphasis on the latest research on IL-17A-mediated inflammatory responses in the CNS. Moreover, it provides an overview of the FDA-approved and investigational monoclonal antibodies and small-molecule inhibitors that target IL-17 and its receptors.

## 2. IL-17A and Neuro-Inflammation

Neurodegenerative diseases have various pathological and clinical manifestations, including selective brain region vulnerability and protein aggregation [27]. In addition to the neuropathological and clinical characteristics of neurodegenerative diseases, there is evidence of persistent and chronic inflammation. Consequently, the neuro-inflammatory response is regarded as a common pathophysiological mechanism [28]. While inflammation is typically neuroprotective, sustained chronic inflammation within the CNS has been linked to neurotoxicity and the development of neurodegenerative diseases [29]. It should be noted that a number of cells are involved in maintaining the homeostasis of the CNS, including microglia, astrocytes, oligodendrocytes, endothelial cells, and pericytes [30]. Neuro-inflammation primarily activates microglia and astrocytes, which play a central role in the innate immune response of the CNS [31]. In conventional classification, these cells are divided into two distinct phenotypes: the M1/M2 (microglia) and A1/A2 (astrocytes) phenotypes are associated with the regional location, type, and stage of neurodegenerative diseases [32]. Microglia and astrocytes perceive external stimuli through surface receptors (TLR, IL17RA/RC, NLRP3, etc.) and secrete pro-inflammatory cytokines, chemokines, lipid mediators, NO, and other substances to recruit additional immune cells and eliminate pathological factors [32]. Nevertheless, the phenotype receptors or inflammatory mediators of microglia and astrocytes can be targeted for glial cell activation. Consequently, sustained inflammatory processes may contribute to the development and progression of neurodegenerative disorders [5]. In recent years, there has been a growing interest in the potential role of T helper cell 17 (Th17) and IL-17A in the pathogenesis of neurodegenerative diseases. It has been demonstrated that IL-17A plays a significant role in neuro-inflammation. It is produced by various immune and CNS-resident cells. IL-17A induces glial cell activation, triggers inflammatory cascades, and compromises BBB integrity [33]. The following sections discuss the production and release of IL-17A (Section 2.1), its impact on glial cell polarization and activation (Section 2.2), and its role in BBB disruption, which facilitates immune cell infiltration into the CNS (Section 2.3).

### 2.1. Induced Release of IL-17A

**Figure 1:**
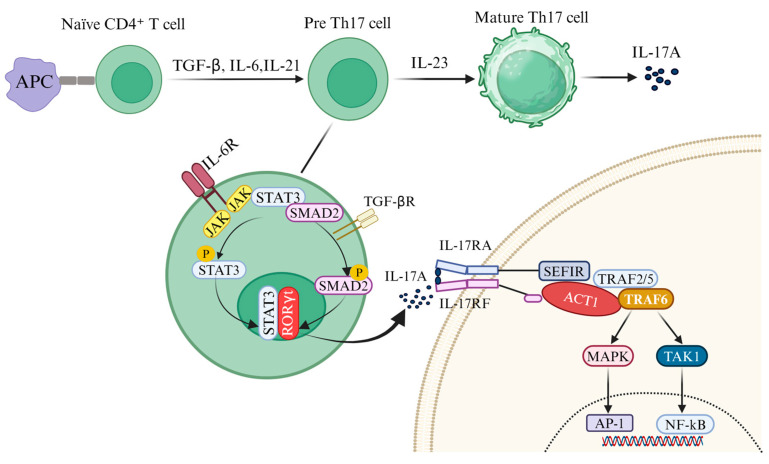
Differentiation of T helper (Th17) Cells and Interleukin-17A (IL-17A) signaling pathway. Th17 cells arise from the differentiation of naïve cluster of differentiation 4^+^ (CD4^+^) T cells following stimulation by antigen-presenting cells (APCs). This process is driven by the combined action of interleukin-6 (IL-6)and transforming growth factor-beta (TGF-β). IL-6 binds to its receptor (IL-6 receptor, IL-6R), activating the Janus kinase 2/signal transducer and activator of transcription 3 (JAK2/STAT3) signaling pathway and resulting in the phosphorylation of STAT3. Simultaneously, TGF-β engages its receptor to induce the phosphorylation of SMAD2. Once phosphorylated, STAT3 (p-STAT3) and SMAD family member 2 (SMAD2; p-SMAD2) translocate to the nucleus, where they cooperate with the transcription factor retinoic acid receptor-related orphan receptor gamma t (RORγt) to bind the IL-17 promoter and initiate IL-17A transcription. Upon secretion, IL-17A binds to its receptor complex (IL-17RA/IL-17RC), triggering downstream signaling cascades. The intracellular similar expression to fibroblast growth factor genes/IL-17 receptor (SEFIR)domain of the receptor interacts with the SEFIR motif on the nuclear factor kappa B activator 1 (Act1) adaptor protein. Act1 then recruits tumor necrosis factor receptor-associated factor 6 (TRAF6) and TRAF2/5, which bind to its TRAF-binding sites, activating the classical nuclear factor kappa B (NF-κB) and MAPK signaling pathways. These pathways collectively amplify the pro-inflammatory responses mediated by IL-17A. These signaling cascades ultimately contribute to glial cell activation and BBB disruption, key processes in IL-17A-mediated neuro-inflammation.

Cluster of differentiation 4^+^ (CD4^+^) T helper (Th) cells play an essential role in the adaptive immune system, serving as the key mediators of immune responses. Naïve CD4^+^ T cells become activated when antigen-presenting cells (APCs) present an antigen. This activation initiates a rapid process of proliferation and differentiation, leading to the formation of distinct Th subsets, including Th1, Th2, Th17, and regulatory Treg cells [34]. It has been discovered that naive T cells can differentiate into a novel subset, Th17 cells, under the combined influence of transforming growth factor-beta (TGF-β) and IL-6. Th17 cells are defined by their expression of the transcription factor retinoic acid receptor-related orphan receptor gamma t (RORγt) and their production of a unique repertoire of pro-inflammatory cytokines, including IL-17A, IL-17F, IL-21, IL-23, IL-3, interferon-gamma (IFNγ), and granulocyte–macrophage colony-stimulating factor (GM-CSF) [35] (Figure 1).

The differentiation and maturation of Th17 cells occur through three well-defined stages [36]. In the initial stage, the binding of TGF-β, IL-6, or IL-21 to their respective receptors triggers the differentiation of naïve CD4^+^ T cells into Th17 precursor cells, with IL-6 playing a central role [37]. Notably, IL-21 can serve as an alternative pathway to IL-6. During the second stage, IL-21, secreted by Th17 cells in an autocrine feedback loop, is critical for driving differentiation, promoting proliferation, and inducing the expression of the IL-23R [38]. In the final stage, IL-23 sustains Th17 differentiation, facilitating the robust production of the pro-inflammatory cytokine IL-17A [39]. This intricate differentiation process is regulated by multiple cytokine-activated signaling pathways [33]. IL-6 binds to its receptor (IL-6R) to activate the JAK2/STAT3 pathway, leading to the nuclear translocation of STAT3 and the subsequent upregulation of the transcription factors RORγt and RORα. Concurrently, TGF-β signaling activates SMAD2 through its receptor, enabling SMAD2 to translocate to the nucleus and initiate the transcription of IL-17A [40].

**Figure 2:**
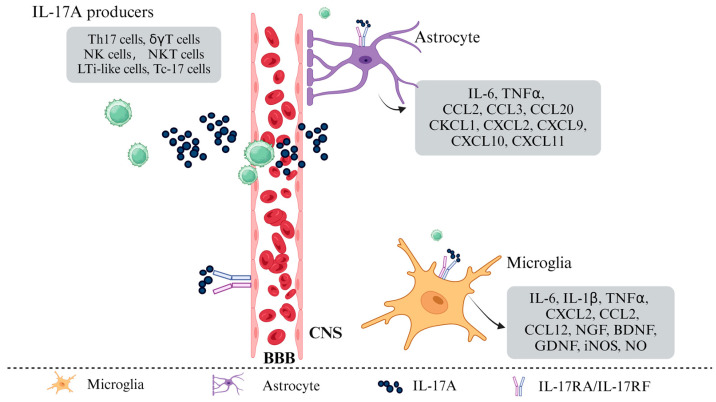
IL-17A-mediated immune responses in the Central Nervous System (CNS). IL-17A, produced by peripheral immune cells (upper left), infiltrates the CNS and is also produced by resident astrocytes and microglia. This cytokine interacts with receptors on various CNS cell types. IL-17A acts on endothelial cells, inducing BBB disruption by downregulating the expression of tight junction and cell adhesion molecules. BBB disruption facilitates the uncontrolled infiltration of peripheral immune cells, including those that produce IL-17A. Signaling of IL-17A in astrocytes and microglia triggers the expression of numerous pro-inflammatory mediators, while also promoting factors that aid in tissue repair and inflammation resolution. IL-17A-induced glial cell activation and BBB breakdown collectively contribute to sustained neuro-inflammation and CNS pathology.

Initially identified as cytotoxic T lymphocyte antigen 8 (CTLA-8), IL-17A was cloned in 1993 and is now recognized as the hallmark cytokine of Th17 cells [41]. The IL-17 receptor was first characterized in 1995, and IL-17A is produced not only by Th17 cells but also by various cell types within the CNS, including γδT cells, TCR-β^+^ T cells, natural killer T (NKT) cells, group 3 innate lymphoid cells (ILC3s), Paneth cells, neutrophils, and microglia [42,43,44] (Figure 2). IL-17A, along with IL-17F and the IL-17A/F heterodimer, signals through a shared receptor complex (IL-17RA/RC), mediating pro-inflammatory effects implicated in numerous pathological conditions. Upon binding to the IL-17RA/RC complex, IL-17A recruits the E3 ubiquitin ligase Act1 through the intracellular SEFIR domain of the receptor. Act1 subsequently engages TRAF6, triggering the downstream activation of the NF-κB and MAPK/AP-1 signaling pathways, thereby amplifying pro-inflammatory responses [45] (Figure 1). IL-17A acts on multiple cell types in the CNS, particularly glial cells that play a central role in mediating neuro-inflammation.

### 2.2. IL-17A Induced Polarization of Glial Cells

Once IL-17A is secreted, it interacts with CNS-resident cells, particularly astrocytes and microglia, which express IL-17RA. The binding of IL-17A to its receptor triggers downstream signaling cascades that influence glial cell polarization and function [46]. This process plays a crucial role in shaping the neuro-inflammatory environment. Murine and human astrocytes express IL-17RA, which activates the NF-κB pathway via Act1 when IL-17A binds to IL-17RA [47]. In vitro studies have demonstrated that IL-17A induces a pro-inflammatory state in astrocytes, characterized by the production of multiple cytokines and chemokines, including IL-6, TNF-α, C-C motif chemokine ligand 2 (CCL2), CCL3, CCL20, C-X-C motif chemokine ligand 1 (CXCL1), CXCL2, CXCL9, CXCL10, and CXCL11 (Figure 2). Furthermore, transcripts of these pro-inflammatory mediators are also detectable in astrocytes during experimental autoimmune encephalomyelitis (EAE) [15,48,49]. Additionally, the anti-IL-17A monoclonal antibody secukinumab can inhibit astrocyte activation and reduce IL-6 production in human astrocytes [12]. The downstream function of Act1 in astrocytes has been experimentally modulated. Conditional knockout or the knockdown of Act1 in astrocytes at various stages yields similar results. In mouse models with Act1-deficient neuroectodermal cells, the severity of EAE is significantly reduced. This indicates that astrocytes are key IL-17-responsive cells that play an essential role in disease processes [13]. Thus, IL-17A-mediated signaling within astrocytes contributes to CNS inflammatory processes.

Microglia, the resident immune cells of the CNS, play a role in monitoring and responding to pathological changes in neural tissue. In the event of a CNS injury, they undergo morphological changes, proliferate, and migrate to the lesion site. There, they perform phagocytic functions to clear microbes, protein aggregates, and dead cells, thereby providing neuroprotection [50]. Moreover, microglia secrete a variety of soluble factors, including neurotrophic factors, which contribute to the central nervous system’s immune response. However, the dysregulation of microglial phenotype or function has the potential to contribute to the pathogenesis of neurodegenerative diseases through excessive synaptic loss. Microglia can recognize phosphatidylserine exposed on neurons with tau protein. This recognition initiates a phagocytic response that produces NO and releases opsonin milk-fat-globule EGF-factor-8 (MFGE8) which is a critical molecule in neuron phagocytosis [51]. Furthermore, surface molecules on microglia, such as Leucine-rich repeat-containing protein 33 (LRRC33) and Triggering Receptor Expressed on Myeloid cells 2 (TREM2), regulate cell signaling pathways by interacting with specific proteins [52,53]. These biological processes are of great consequence in the onset and progression of neurodegenerative diseases. The precise mechanisms by which IL-17A contributes to neurodegenerative disease remain under debate. However, there is a consensus that IL-17A has a pro-pathogenic effect in these conditions. It achieves this by activating glial cells, particularly microglia. For example, in a PD model, IL-17A activates microglia in vitro. This activation leads to accelerated death of dopaminergic neurons [24]. Suppression of IL-17RA signaling in microglia eliminates this pro-pathogenic effect of IL-17A, further supporting the pathogenic role of microglia in PD [24]. Moreover, microglia in the CNS of EAE mice exhibit increased IL-17RA expression, possibly induced by Toll-like receptor (TLR) signaling [54]. IL-17A treatment in EAE models can induce microglia to secrete chemokines [49]. In aged rats, IL-17A contributes to lipopolysaccharide (LPS)-induced neuro-inflammation and cognitive impairment by activating microglia [55]. Nevertheless, the precise relationship between IL-17A and microglia in neurodegenerative diseases remains uncertain. For instance, the overexpression of IL-17A in the mouse brain does not result in microglial activation in an AD mouse model [56]. In vitro, the stimulation of microglia with IL-17A has been observed to upregulate the expression of various inflammatory mediators, including IL-6 and CXCL2, as well as neurotrophic factors such as nerve growth factor (NGF), brain-derived neurotrophic factor (BDNF), and glial cell line-derived neurotrophic factor (GDNF) (Figure 2). It is only when microglia have been previously stimulated (for example, by LPS) that IL-17A can enhance inflammation while promoting tissue repair and the resolution of inflammation. This phenomenon has also been observed in non-MS pathologies [57]. These findings suggest that further investigation is warranted to elucidate the mechanisms of IL-17A and microglia interactions in neurodegenerative diseases.

### 2.3. IL-17A Mediated BBB Disruption

Beyond its role in glial cell activation, IL-17A also contributes to neuro-inflammation by disrupting BBB integrity. This disruption allows peripheral immune cells to infiltrate, exacerbating CNS inflammation. Th17 cells are implicated in CNS dysfunction and disease activity in conditions like relapsing–remitting multiple sclerosis (RRMS) and EAE [58]. In vitro studies show that human Th17 cells across the BBB more efficiently than other T cell subsets and can exert neurotoxic effects, indicating their pivotal role in the onset and progression of MS and EAE [59]. Peripherally, Th17 cells activate bone marrow neutrophils, leading to immature monocytes in circulation [60]. BBB disruption is critical for MS lesion formation and accumulation. Consequently, increased neutrophil recruitment, along with heightened protease activity, attracts further macrophage and monocyte migration, triggering neuro-inflammation and causing sustained myelin and axon damage [61,62]. Th17 cells can transition between phenotypes, particularly via transcription factors T-bet and RORγt [63]. Some Th17 cells initially secreting IL-17 can convert to produce interferon-gamma (IFN-γ). Although IL-23 is involved in this reprogramming, evidence shows that IL-17-producing Th17 cells primarily differentiate under TGF-β and IL-6 influence, reducing the reliance on the IL-23 pathway. In a mouse model, Estelle Bettelli and colleagues demonstrated that IL-23 combined with IFN-γ or TGF-β and IL-6 does not significantly affect Th17 differentiation or IL-17 production. Instead, IL-23 enhances sustained IL-17 production by the existing Th17 cells [64]. TGF-β and IL-6 promote Th17/IL-17 production alone [65], but their combined action significantly increases the IL-17-secreting Th cell population. This suggests that the acute release of pro-inflammatory cytokines like IL-6 influences Th17 and regulatory Treg cell development [66]. Yang et al. further described the role of IL-6 and TGF-β in IL-23-dependent pathways, especially in encephalitis. Although Th17 cells differentiate and activate in response to IL-6 and TGF-β, these newly generated Th17 subsets cannot cross the BBB into the CNS. This destabilizing phenotype is characteristic of encephalitis caused by the transcription factor T-bet (rather than cytokine secretion) [67]. Some more aggressive Th17 cells express IL-17A and IFN-γ, displaying features of both Th1 and Th17 cells [68,69]. In summary, IL-17A significantly impacts BBB integrity and monocyte migration, enhancing inflammatory cell recruitment, and compromising BBB structure. This disruption increases protease activity, initiating CNS inflammatory responses.

## 3. IL-17A and Neuro-Inflammation Diseases

IL-17A has emerged as a key player in neuro-inflammatory processes, contributing to the progression of neurodegenerative diseases and CNS BBB disorders. Recent studies highlight its role in promoting neuro-inflammation by acting on resident cells within the CNS, which can exacerbate conditions such as AD, PD, MS, and ALS [20].

### 3.1. IL-17A and MS

Among several CNS inflammatory diseases, MS is most closely associated with Th17 cells and IL-17A, particularly in the EAE murine model. There is a significant correlation between disease severity/progression and these factors. Although the role of IL-17A and IL-17A-producing cells in MS and EAE has been extensively discussed, the role of IL-17 itself has been debated since the discovery of the IL-23/Th17 axis [70,71,72,73,74]. However, strong evidence indicates that IL-17A is involved in the pathogenesis of MS and EAE [75].

Previous research has shown that IL-17A levels are elevated in the cerebrospinal fluid of MS patients compared to controls [76,77], along with an increased frequency of IL-17A-producing T cells [78,79,80], and the presence of IL-17A-producing cells in the CNS [69,74,81]. IL-17A and IL-22 receptors are expressed on endothelial cells in MS lesions. These cytokines can compromise the BBB, and Th17 cells can cross it [58]. Th17 cells have been observed in damaged spinal cords in EAE, forming immune synapses with neurons [82]. In particular, the direct involvement of IL-17A in this neuroimmune interface remains to be confirmed. Previous mechanistic observations have been corroborated by in vitro studies showing that Th17-induced neuronal damage is mediated by a detrimental increase in intracellular Ca2^+^ levels [82]. Indeed, basal IL-17RA expression has been detected in several neuronal populations [83]. Evidence suggests that pathological conditions, such as focal cortical dysplasia, can lead to a significant upregulation of IL-17RA expression [84]. A particularly vulnerable target for IL-17A-mediated neuronal damage is neural stem cells (NSCs) within the subventricular and subgranular zones of the hippocampus. While IL-17A does not induce apoptosis in cultured NSCs, it significantly inhibits neurosphere growth [85]. IL-17A also suppresses NSCs proliferation in a p38 MAPK-dependent manner and inhibits NSCs differentiation, particularly toward astrocytic and NG2-positive lineages [85]. However, IL-17A exhibits neurotrophic properties in the sympathetic neurons of the mesenteric ganglion, promoting neurite outgrowth and acting as a chemotactic gradient. This effect is mediated by NF-κB signaling and occurs independently of glial support [83]. Given the significant heterogeneity among neuronal populations, the effects mediated by IL-17A may vary widely. Therefore, therapeutic interventions targeting IL-17A should be carefully considered.

During EAE progression, T cell infiltration into the CNS occurs in two waves: an initial CCR6-CCL20-dependent entry via the choroid plexus, followed by a second wave crossing CNS blood vessels [86]. The BBB, composed of endothelial cells (ECs), pericytes, and astrocytes, interacts with Th17 cells and their products. While astrocytes respond to IL-17A, their role in BBB function remains unclear [87]. ECs exhibit low baseline IL-17RA expression, which increases in active MS lesions [88]. IL-17A disrupts BBB integrity by downregulating tight junction proteins and increasing permeability. It promotes neuro-inflammation through the production of IL-6, CCL2, and CXCL8. These effects have been validated in vitro using human and mouse BBB models [59].

IL-17A compromises the BBB by downregulating tight junctions, increasing adhesion expression, and promoting monocyte migration [88]. The pharmacological inhibition of the IL-17 pathway mitigates these effects and alleviates EAE symptoms by reducing lymphocyte and monocyte infiltration [89]. These findings highlight the role of the IL-17 pathway in MS and EAE. Although IL-17A-neutralizing antibodies like secukinumab, ixekizumab, and brodalumab are approved for psoriasis treatment, only secukinumab has been clinically tested for MS [90]. In a Phase II trial involving 38 relapsing-remitting MS patients, treatment with secukinumab (10 mg/kg for 24 weeks) did not reduce active brain lesions or relapse rates. Adverse events included a slightly higher infection rate, with one fungal infection reported [90]. Due to these negative results, its development for MS was discontinued. However, it has been used in isolated cases of MS patients with concomitant psoriasis [91]. In these cases, the treatment improved psoriasis but did not control neuro-inflammation, suggesting that different pathways may underlie different autoimmune diseases in the same patient.

### 3.2. IL-17A and AD

AD is the most common neurodegenerative disease causing cognitive impairment in the elderly [92,93]. The histopathologic hallmark of Alzheimer’s disease is the presence of amyloid plaques in the brain, composed primarily of fibrillar β-amyloid peptide-40 (Aβ-40) and β-amyloid peptide-42 (Aβ-42) [94]. The sequential cleavage of amyloid precursor protein (APP) results in the fibrillar forms of amyloid-β found in these plaques [95,96]. High levels of insoluble Aβ peptides in the CNS are critical in AD pathogenesis. These peptides activate the complement pathway [97] and stimulate microglia to produce pro-inflammatory cytokines and chemokines, thereby recruiting inflammatory cells to the CNS [98,99]. Studies suggest that plasma IL-17 levels may serve as a biomarker to distinguish AD patients from cognitively healthy individuals, and that cerebrospinal fluid IL-17 levels may help identify tau pathology in frontotemporal dementia (FTD) [100,101]. Activated Th17 cells within the CNS can release pathogenic cytokine IL-17A, recruit neutrophils, and amplify inflammatory cascades, thus promoting neuro-inflammation and neurodegeneration in AD [102,103]. Genetic variations that upregulate the expression of IL-17A are strongly linked to AD pathogenesis. Growing evidence suggests that IL-17A contributes to neuronal degeneration in AD by interacting with Aβ, activating microglia, disrupting the BBB, and promoting systemic neuro-inflammation [104].

The microglia-mediated pro-inflammatory process contributes to neurodegeneration [98,105]. However, microglia also exhibit a protective function by phagocytosing Aβ aggregates [106]. Aβ peptides induce the microglia production of reactive nitrogen and oxygen species, leading to oxidative stress. This, in turn, stimulates Th17 cell production and IL-17 secretion [102,107]. IL-17 primarily recruits and stimulates neutrophils in AD pathogenesis. Studies show that in mice overexpressing human mutant APP, Aβ aggregates are crucial for recruiting neutrophils into the CNS. These aggregates promote chemotaxis, stimulate IL-17 production, and enhance neutrophil infiltration into the CNS [102] despite lower IL-17A levels in their mesenteric lymph nodes due to reduced Th17 differentiation [108]. Neutrophils are a primary target and source of IL-17A in the CNS, potentially playing a pivotal role in AD pathology by promoting inflammation and tissue damage. In vitro studies suggest that IL-17A may induce neuronal autophagy, resulting in neurodegeneration [109].

In AD, while the innate immune role is well studied, recent triple-transgenic mouse model findings show significant adaptive immune activation, including T and B lymphocytes [110]. These lymphocytes produce more cytokines such as IL-2, TNF-α, IL-17, and GM-CSF, hinting at Th17 cell-driven neurodegeneration [110]. Following intrathecal Aβ-42 peptide administration, rats showed an upregulated expression of IL-17, IL-22, and RORγt. The same study found that Aβ-42 disrupted the BBB, aiding Th17 cell brain infiltration [111]. In a separate study, Tian et al. observed that postoperative cognitive dysfunction correlates with elevated IL-17A levels in the hippocampus, implicating IL-17A-mediated hippocampal damage in Aβ1-42 accumulation and the subsequent impairment of cognitive functions [112]. Aβ-42 injected rats also had raised brain RORγt, IL-23, and IL-17, alongside lower Treg-related cytokines like TGF-β and IL-35 [113].

In the brain, activated Th1 and Th17 cells release IFN-γ and IL-17A, worsening inflammation, immune cell activation, and AD neuropathology [103]. In the context of MS, Th17 cell-derived cytokines bind neuronal receptors, triggering apoptotic neurodegeneration [114]. Notably, AD model rats had elevated Fas and FasL suggesting that Th17 cells drive neuronal apoptosis via Fas/FasL-mediated pathways [111,115,116]. Furthermore, in TLR4-mutated AD mice, high brain IL-1β levels correlated with increased IL-17A, linking innate immune dysfunction to Th17-mediated neuro-inflammation [117]. A recent study demonstrated that anti-IL-17A antibody treatment improved cognition and reduced neuro-inflammation in Aβ1-42 injected mice, cutting Aβ1-42, GFAP, S100 protein, and various inflammatory mediators and cytokines, underscoring IL-17A’s pathological role [118]. These support IL-17A’s involvement in AD-related neuro-inflammation and neurodegeneration [119].

However, some studies suggest IL-17A’s protective role in AD models. For instance, the intracranial overexpression of IL-17A has been shown to mitigate cerebral amyloid angiopathy and improve anxiety and learning deficits [120]. ICR mice given IL-17A exhibited better spatial learning linked to neural precursor cell maturation and neural progenitor cell proliferation suppression [120]. In humans, AD patients display elevated numbers of CD4^+^ and CD8+ T cells within the brain parenchyma and vascular endothelium compared to healthy controls [121]. Furthermore, T lymphocytes from AD patients produce more Th17-related cytokines like IL-21 and RORγt, while monocytes make more IL-6 and IL-23 [122]. Mild cognitive impairment (MCI) due to AD pathology has a higher peripheral blood Th17 cell proportion than non-AD or healthy individuals [123]. AD patients also have significantly higher serum IL-17 and IL-23 levels than healthy controls (*n* = 212), with IL-17A a potential plasma biomarker for AD [100]. Additionally, cerebrospinal fluid IL-17A levels have been proposed as a potential tool for the pre-mortem identification of tau pathology in frontotemporal dementia, further emphasizing the clinical relevance of this cytokine [101].

An ideal AD vaccine has been proposed to promote a Th2-skewed immune response and suppress Th1 and Th17 responses to Aβ, thereby reducing neuro-inflammation and preventing neurodegeneration [124]. Although evidence highlights IL-17A’s role in AD, the precise mechanisms driving its upregulation its CNS upregulation in AD patients remain unclear. One plausible hypothesis is that microbial infections, such as respiratory infections [125] or gut immune surveillance [108], may trigger excessive CNS IL-17A, aiding Aβ deposition. Alternatively, poor Aβ clearance might active innate immune receptors, driving IL-17A production and sustaining AD’s pathological cycle.

### 3.3. IL-17A and PD

PD is a neurodegenerative disorder characterized by the progressive degeneration of midbrain neurons (MBNs), particularly the dopaminergic (DA) neurons in the substantia nigra pars compacta (SNpc). Hallmarks of the disease include Lewy body formation, intracellular aggregation of α-synuclein (α-syn), and the involvement of neuro-inflammation [126]. Previous studies have shown that in patients with PD, there is a higher number of cells that produce IL-17A [127]. A research system has highlighted the function of human T lymphocytes. In sporadic PD, these T lymphocytes can cause cell death by making human iPSC-derived midbrain neurons (MBNs) die [128]. Studies have shown that the frequency of Th17 cells is significantly higher in the peripheral blood of PD patients [127]. Additionally, post-mortem brain tissue from these patients exhibited an increased number of T lymphocytes. When PD iPSC-derived MBNs were co-cultured with T lymphocytes or exposed to IL-17A, they showed enhanced neuronal death due to the upregulation of IL-17R and the activation of NF-κB. Blocking IL-17A or IL-17R, or using the anti-IL-17A antibody secukinumab, rescued these neurons from death. These findings indicate that IL-17A-producing T lymphocytes play a critical role in sporadic PD [128]. Zhan Liu and colleagues explored the role of IL-17A in a mouse model of PD, demonstrating that IL-17A exacerbates neuro-inflammation and neurodegeneration through microglial activation. The binding of IL-17A to IL-17RA receptors on microglia induces the release of pro-inflammatory factors, further promoting the damage and death of dopaminergic neurons [24]. Consequently, blocking IL-17A signaling effectively reduces neuro-inflammation, underscoring the therapeutic potential of targeting IL-17A in PD.

### 3.4. IL-17A and ALS

Recent findings indicate that IL-17A plays a crucial role in the immune-related processes underlying ALS. Higher levels of IL-17A and IL-23 have been detected in both CSF of ALS patients compared to individuals with non-inflammatory neurological conditions. This suggests Th17 cell activity in ALS [129]. Notably, these cytokine levels did not correlate with disease duration, disability scale, or clinical subtypes. This implies a constant inflammatory state that is not tied to disease progression [129]. The shift toward pro-inflammatory responses in ALS patients, marked by increased Th1 and Th17 cells and reduced Th2 and regulatory T cells, further supports the involvement of IL-17A in neuro-inflammation [130].

From a mechanistic standpoint, IL-17A-expressing CD8+ T cells and mast cells infiltrate the spinal cords of ALS patients. They cluster with macrophages that produce IL-1β and TNF-α, worsening neuro-inflammation [131]. Additionally, mononuclear cells exposed to aggregated superoxide dismutase-1 (SOD1), a protein implicated in ALS pathology, show higher expression of IL-1β, IL-6, IL-23, and IL-17A. This suggests that misfolded SOD1 may trigger chronic inflammation driven by IL-17A [132]. Given these findings, targeting IL-17A and its regulators may offer a promising treatment strategy. It may help control inflammation and reduce nerve cell damage in ALS.

## 4. IL-17A as a Therapeutic Target

IL-17A is secreted by diverse adaptive and innate immune cells, including Th17 cells, CD8^+^ T cells, γδ T cells, natural killer T (NKT) cells, and innate lymphoid cells [133]. This cytokine is crucial for protective immunity against both intracellular and extracellular pathogens. It also contributes to the progression of inflammatory responses. Dysregulated Th17 cell activity and increased IL-17A levels are involved in the pathogenesis of autoimmune diseases characterized by chronic inflammation, including psoriasis (PsO), psoriatic arthritis (PsA), MS, and rheumatoid arthritis (RA) [134]. Therapeutic strategies targeting IL-17A, such as monoclonal antibodies (mAbs) that neutralize the cytokine or its receptor, have shown promising results in immune-mediated diseases. However, further research is needed to fully understand their long-term safety and broader clinical implications. In recent years, several IL-17 inhibitors have gained regulatory approval, including ixekizumab, secukinumab, brodalumab, Netakimab, and bimekizumab [135,136,137]. Secukinumab and ixekizumab specifically target IL-17A, while brodalumab inhibits IL-17RA. Secukinumab has received U.S. FDA approval for the treatment of plaque psoriasis, PsA, ankylosing spondylitis (AS), and non-radiographic axial spondyloarthritis (nr-axSpA) [138,139]. Brodalumab, meanwhile, is approved for patients with moderate-to-severe plaque psoriasis who have not responded adequately to topical or systemic therapies [140]. Clinical trials have shown that secukinumab, ixekizumab, and brodalumab are effective in managing PsO in the short term. Their safety profiles are comparable to other FDA-approved biologic therapies. However, their long-term safety is still being evaluated [141]. Netakimab, an IL-17A-targeting agent, has been approved in Russia primarily for the treatment of PsO [142,143]. Bimekizumab, a dual inhibitor of IL-17A and IL-17F, is currently undergoing clinical trials for indications such as psoriasis, PsA, and ankylosing spondylitis [144,145,146] (Table 1).

Biologics are therapeutic agents that are genetically engineered from biomolecules like proteins and nucleic acids. mAbs are generally well tolerated, but they can cause serious, rare, and unpredictable adverse drug reactions [147]. FDA-approved IL-17A inhibitors are predominantly monoclonal antibodies. They have demonstrated clinical efficacy but also carry potential risks and long-term challenges. IL-17A plays a crucial role in mucosal immunity, especially in defending against fungal and bacterial infections. As a result, patients receiving IL-17A inhibitors have an increased risk of opportunistic infections, such as cutaneous and mucosal candidiasis [148]. Emerging data also suggest a possible link between IL-17A blockade and alterations in tumor immune surveillance, although definitive evidence is still lacking [149]. Additionally, some patients experience disease rebound or loss of efficacy after stopping IL-17A-targeted therapy. This means they need to adhere to long-term treatment [150]. While mAbs are the main therapeutic strategy for IL-17A inhibition, there is growing interest in small molecule inhibitors that modulate the IL-17A signaling pathway. These alternatives may have advantages such as oral administration and broader regulatory effects on IL-17A-associated pathways. This could help address some of the long-term concerns associated with monoclonal antibody therapy.

In addition to the monoclonal antibodies targeting IL-17A, research on small molecule inhibitors regulating the IL-17A signaling pathway has also increased. Aeri Park et al. reported that celastrol significantly reduced pro-inflammatory cytokines (such as IL-6 and TNF-α) by directly inhibiting the IL-17A signaling pathway. It also regulated the autophagy process through the IL-17A pathway. It has important application prospects in the treatment of inflammatory diseases like psoriasis [151]. Dou et al. demonstrated that resveratrol ameliorates intestinal epithelial and vascular permeability caused by transient middle cerebral artery occlusion (tMCAO). This protective effect was achieved by modulating the intestinal microbiota. This, in turn, reduces the accumulation of Th17 cells in the enteric plexus and decreases IL-17A levels in serum and brain tissue [152]. Salidroside (Sal) has been shown to reduce the expression of RORγt in peripheral circulation, decrease the number of Th17 cells, and increase the population of peripheral Treg cells in ischemic brain tissue. Under hypoxic conditions, Sal significantly inhibits the expression of pro-inflammatory factors, including IL-6, TNF-α, MCP-1, STAT3, and NF-κB proteins in Th17 and Treg cells [153]. Hyperforin, a bioactive component of *Hypericum perforatum*, protects against acute ischemic stroke by reducing IL-17A expression and inhibiting IL-17A-mediated microglial activation. This leads to decreased infarct volume, improved neurological outcomes, and a shift from M1 to M2 microglial phenotypes, highlighting its potential as a neuroprotective agent [154]. Furthermore, matrine has shown neuroprotective potential in in vivo models of AD. In Aβ1-42-induced AD model rats, matrine significantly improved cognitive deficits, as shown by better learning and memory. These effects were due to its ability to modulate the cytokine profile of Th17 and Treg cells, particularly by reducing pro-inflammatory IL-17A levels and increasing anti-inflammatory TGF-β levels [155]. Jun Wang et al. reported that huperzine A improved clinical symptoms and neurodegeneration in EAE mice by inhibiting neuro-inflammation driven by pro-inflammatory T cells, including Th1 and Th17 subsets. These findings suggest that huperzine A could be a therapeutic candidate for MS [156].

**Table 1:** Monoclonal antibodies targeting IL-17A/IL-17RA and their clinical indications.

Monoclonal Antibody	Name of Product	On the Target	Clinical Indication	Reference
Secukinumab	Cosentyx	IL-17A	Plaque psoriasis; ankylosing spondylitis; psoriatic arthritis	[157,158,159]
Ixekizumab	Taltz	IL-17A	Plaque psoriasis; psoriatic arthritis; ankylosing spondylitis; Non-radiographic axial spondyloarthritis	[160,161,162]
Brodalumab	Siliq	IL-17RA	Moderate-to-severe plaque psoriasis	[163,164,165]
Netakimab	Efleira	IL-17A	Psoriasis	[142,143]
Bimekizumab	Phase Ⅲ clinical trial	IL-17A/IL17F	Psoriasis; psoriatic arthritis; ankylosing spondylitis	[145,146,166,167,168]

## 5. Conclusions

Neurodegenerative diseases severely affect individuals and disrupt daily life. Inflammation is a major contributor to neurodegeneration, with pro-inflammatory cytokines being of particular concern. Targeting IL-17A is, therefore, highly relevant therapeutically for treating these diseases. This review highlights the critical role of neuro-inflammation in the development of neurodegeneration. It focuses on IL-17A from Th17 cells. Under pathological conditions, IL-17A crosses the BBB, acts on glial cells, and contributes to neurodegenerative processes. Secukinumab, a monoclonal antibody targeting IL-17A, has shown efficacy in treating MS. IL-17A is also a key regulator of neuro-inflammation in other neurodegenerative diseases, such as AD and PD. Elevated IL-17A levels in the cerebrospinal fluid and peripheral blood activate microglia and astrocytes. This triggers inflammatory cascades that worsen neuronal damage. Targeting IL-17A in drug discovery and development holds promising therapeutic potential. Inhibiting IL-17A signaling may reduce neuro-inflammatory responses and thereby protect neurons from inflammation-related injury. Furthermore, IL-17A-targeted therapies could restore BBB integrity, prevent the infiltration of inflammatory mediators, and slow disease progression.

Although the current evidence suggests that IL-17A inhibition holds promise for treating neurodegenerative diseases, further exploration is needed in several key areas. Most studies on IL-17A-targeted therapies have been short-term. There is limited data on the long-term effects of IL-17A inhibition in these neurodegenerative diseases. Future research should focus on long-term safety profiles. This includes potential risks such as infections and immune dysregulation, especially in vulnerable groups like the elderly or those with comorbidities. While IL-17A’s role in neuro-inflammation is clear, its exact mechanisms in the pathophysiology of different neurodegenerative diseases are still unclear. Further studies should investigate how IL-17A interacts with other inflammatory pathways. Also how blocking it affects neuro-inflammatory cascades at the molecular level needs to be explored. Identifying reliable biomarkers that predict response to IL-17A-targeted therapies would greatly enhance personalized treatment strategies. Future studies should focus on finding these biomarkers, such as IL-17A levels in cerebrospinal fluid or peripheral blood, and studying their predictive value for treatment effectiveness. At present, although IL-17A-targeted therapies are primarily focused on mAbs, research has increasingly explored small-molecule inhibitors, especially those derived from traditional herbal medicines. These inhibitors may have distinct advantages, such as oral administration and a broader influence on inflammatory pathways. However, the development of specific small-molecule inhibitors remains limited. This highlights the need to prioritize research on their synthesis, efficacy, and safety.

In conclusion, IL-17A plays an important role in the pathophysiology of neurodegenerative diseases. Targeting IL-17A could offer novel therapeutic strategies and interventions for managing these conditions. This review highlights the significance of IL-17A in neuro-inflammation and provides insights into drug screening for potential IL-17A-targeted treatments in neurodegenerative diseases. In the future, IL-17A-targeted therapies, whether monoclonal antibodies or small-molecule inhibitors, could be integrated into personalized treatment for diseases that currently limited treatment options.
